# Analysis of zwitterionic and anionic N-linked glycans from invertebrates and protists by mass spectrometry

**DOI:** 10.1007/s10719-016-9650-x

**Published:** 2016-02-22

**Authors:** Katharina Paschinger, Iain B. H. Wilson

**Affiliations:** Department für Chemie, Universität für Bodenkultur, 1190 Wien, Austria

**Keywords:** Glycomics, Mass spectrometry, Phosphate, Sulphate, Glucuronate, Phosphorylcholine, Phosphoethanolamine, Aminoethylphosphonate

## Abstract

Glycomic analyses over the years have revealed that non-vertebrate eukaryotes express oligosaccharides with inorganic and zwitterionic modifications which are either occurring in different contexts as compared to, or are absent from, mammals. Examples of anionic N-glycans (carrying sulphate or phosphate) are known from amoebae, fungi, molluscs and insects, while zwitterionic modifications by phosphorylcholine, phosphoethanolamine and aminoethylphosphonate occur on N-, O- and lipid-linked glycans from trichomonads, annelids, fungi, molluscs, insects, cestodes and nematodes. For detection of zwitterionic and anionic glycans, mass spectrometry has been a key method, but their ionic character affects the preparation and purification; therefore, as part of a glycomic strategy, the possibility of their presence must be considered in advance. On the other hand, their ionisation and fragmentation in positive and negative ion mode mass spectrometry as well as specific chemical or enzymatic treatments can prove diagnostic to their analysis. In our laboratory, we combine solid-phase extraction, reversed and normal phase HPLC, MALDI-TOF MS, exoglycosidase digests and hydrofluoric acid treatment to reveal N-glycans modified with anionic and zwitterionic moieties in a wide range of organisms. It is to be anticipated that, as more species are glycomically analysed, zwitterionic and anionic modifications of N-glycans will prove rather widespread. This knowledge is - in the longer term - then the basis for understanding the function of this cornucopia of glycan modifications.

## Introduction

**Fig. 1:**
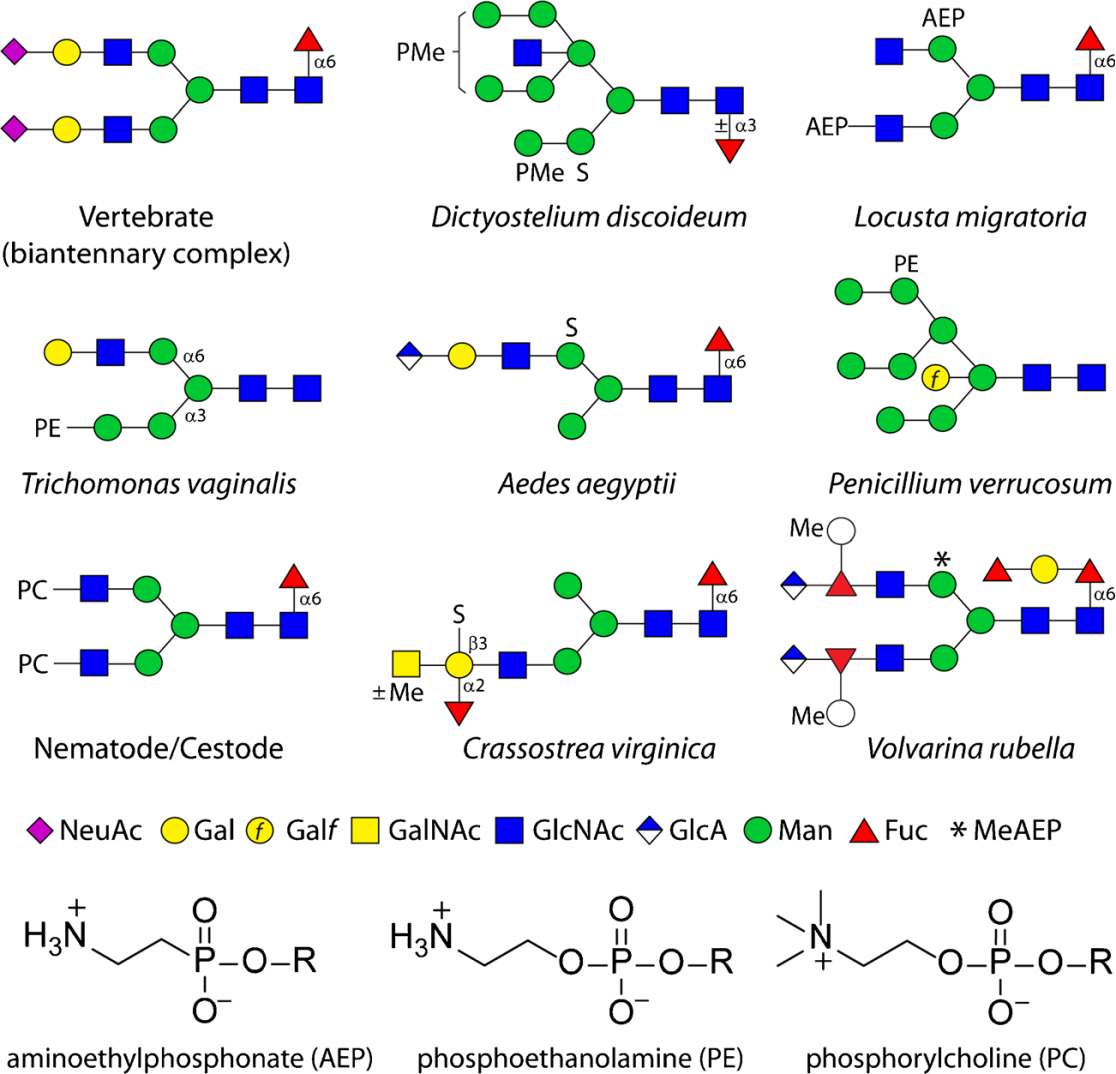
Example anionic and zwitterionic N-glycans from non-mammalian sources. Glycans are shown according to the nomenclature of the Consortium for Functional Glycomics [11] as compared to a biantennary sialylated vertebrate N-glycan (all sugars are pyranose, except for Gal*f*, galactofuranose). AEP, aminoethylphosphonate; PC, phosphorylcholine; PE, phosphoethanolamine; PMe, methylphosphate; S, sulphate; *, *N*-methylaminoethylphosphonate. The chemical structures of the zwitterionic modifications are also shown. The named organisms in which these example glycans are found may not represent the only species to contain these structures, but are the ones in which these are proven [4, 12–19]

Glycoconjugates are not merely the product of ‘ligation’ of an oligosaccharide with the underlying protein or lipid, but can be further modified with non-sugar anionic components such as sulphate and phosphate. Due to their charged character, these modifications have a profound effect on recognition events *in vivo*. For example, mannose-6-phosphate is well known as being necessary for recognition and targetting of mammalian lysosomal enzymes [1], the clearance of mammalian pituitary glycoprotein hormones by Kupffer cells is mediated by lectins binding sulphated GalNAc [2] and the sulpho-glucuronate-based HNK-1 epitope has functions in neural development [3]. However, the biological significance of glycan phosphorylation and sulphation (other than proteoglycans) in non-mammalian systems remains obscure, but this is in part due to a probable underestimation of their occurrence. Nevertheless, previous reports indicate that methyl and mannose phosphodiesters occur respectively, on slime mould and yeast glycoproteins [4, 5] and that sulphate is a component of fucans and galactans from marine organisms [6], invertebrate glycosaminoglycans [7], sea urchin sialoconjugates [8], plant chloroplast sulfoquinovosyldiacylglycerol [9] and *Dictyostelium* N-glycans [4, 10]. More recent data (see also Fig. 1) using mass spectrometry mean that this list can be extended to include sulphate on mollusc and insect N- and O-glycans [12–14].

Additionally, rather well known from mammalian systems are the ‘organic’ anionic modifications of glycans including sialic and glucuronic acids; in protists, the occurrence of sialic acid is probably restricted to trypanosomatids which steal this unit from host glycoproteins via their *trans-*sialidase [20]. In contrast, the presence of sialic acid in insects was, for some years, a controversial topic, but low levels of sialylated N-glycans and a relevant sialyltransferase were found in *Drosophila* [21, 22]. Sialylation may have roles in neural development in *Drosophila* [23] and fertilisation of sea urchins [24]. Glucuronic acid, on the other, hand is present as a component of chondroitin and heparan sulphates from all animals, including invertebrates [25] but is now known to be present on insect N-, O- and lipid-linked glycans [12, 26, 27]; it has been recently found (also in methylated form) on the antennae of N-glycans of two mollusc species [14, 28]. On the other hand, glucuronic acid (but not sialic acid) has been found on O-glycans from both *Schistosoma mansoni* and *Caenorhabditis elegans* [29, 30], but neither have been detected, to date, as modifications of nematode or trematode N-linked glycans.

In terms of the zwitterionic modifications, phosphorylcholine and phosphoethanolamine are of immunological relevance as targets of mammalian pentraxins (*e.g*., C-reactive protein) and antibodies [31, 32]; furthermore, phosphorylcholine is an immunomodulatory modification of N-glycans and glycolipids from nematode parasites which may interact directly or indirectly with Toll-like receptors of immune cells and so effect the balance of host immune systems [33]. However, other than the phosphoethanolamine on glycosylphosphatidylinositol anchors [34], zwitterionic modifications of glycans are unfamiliar to those who specialise in mammalian glycomics. In ‘lower’ organisms (for some examples, see Fig. 1), the zwitterion phosphorylcholine has been reported as a phosphodiester modification of fungal cell walls [35], of nematode and cestode N-glycans [15, 36, 37] and of glycolipids from fungi, annelids and nematodes [38–40], whereas phosphoethanolamine, the non-methylated version of phosphorylcholine, is present on trichomonad and fungal N-glycans as well as on insect O-glycans and glycolipids [16, 17, 41, 42]. The ‘variant’ of phosphoethanolamine lacking the oxygen ‘bridge’, thereby possessing a C-P bond, is known as aminoethylphosphonate and has been detected (also in methylated form) on mollusc, cnidarian and locust glycoconjugates [14, 18, 43, 44].

For glycan analysis, the presence of anionic moieties is known to pose problems, as their physicochemical properties alter characteristics during purification or result in altered ionisation [45]. Some of the same problems and solutions also apply to zwitterionically-modified oligosaccharides as their polarity means that such glycans can be lost upon organic or solid phase extraction. Also, other than well-characterised sialidases and phosphatases, there are no (pure) enzymes known to cleave any of these modifications, but some chemical treatments (hydrofluoric acid or methanolic HCl) are possible. Certainly, a pre-requisite for understanding the evolution and function of anionic and zwitterionic glycans in non-vertebrate systems is the adequate detection and structural elucidation of the relevant glycomodifications, which have variable effects on chromatographic separations or are of low abundance leading to them being easily overlooked; here, we summarise ways to analyse such glycans in non-mammalian systems, also in a historical context, but with a focus on N-glycomic methodologies used in our laboratory. Naturally, such approaches are also applicable to, or in part first used with, anionic glycans from vertebrates.

## Purification of zwitterionic and anionic glycans

**Fig. 2:**
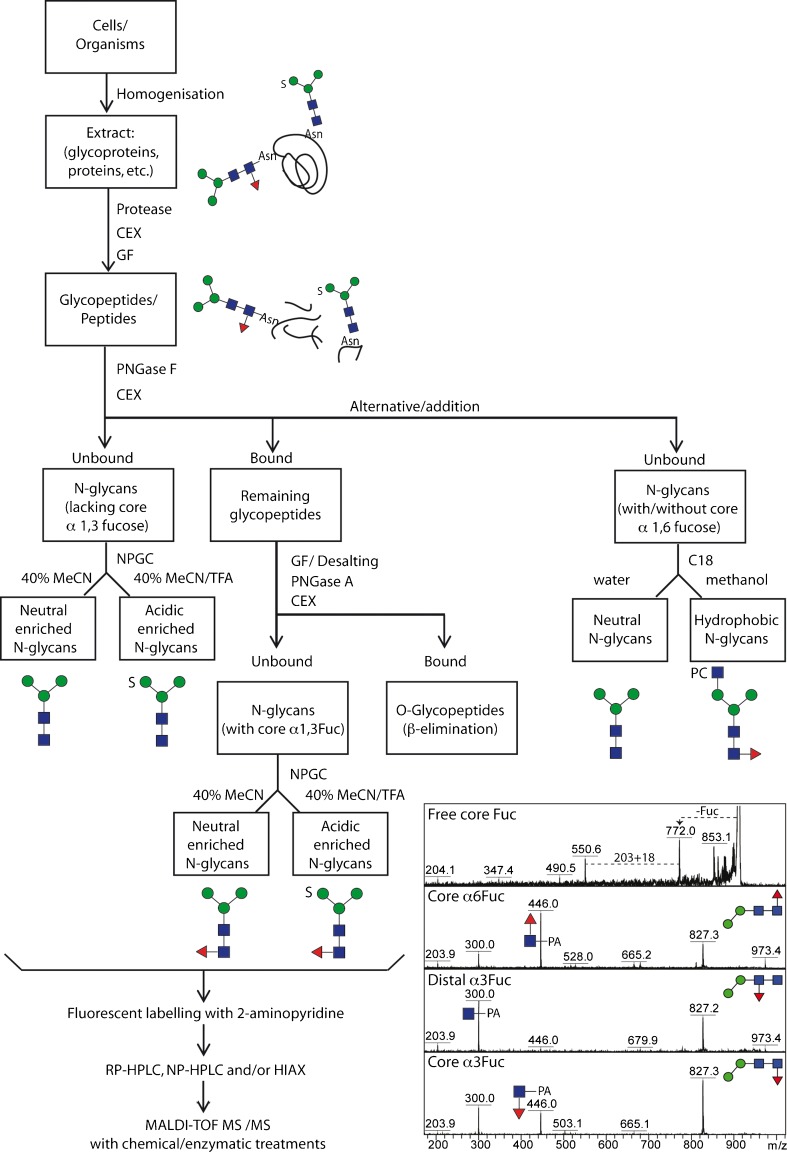
Analytical workflow for off-line LC-MALDI-TOF MS glycan analysis. The overall concept of our N-glycome analytical studies; initially, samples are proteolysed, the glycopeptides enriched by cation exchange (*CEX*) and gel filtration (*GF*) and the glycans released enzymatically, whereby PNGase A (and not PNGase F) is capable of releasing the core α1-3-fucosylated N-glycans. Subsequent sub-fractionation by non-porous graphitised carbon (*NPGC*) and/or reversed-phase (C18) resins result in pools differing in terms of anionic and zwitterionic modifications. Finally, all N-glycans are analysed by different types of HPLC (reversed or normal phase or hydrophilic interaction/anion exchange; RP, NP or HIAX) in combination with MALDI-TOF MS/MS and chemical/enzymatic treatments. The inset shows a comparison of MS/MS of free and pyridylaminated forms of Hex_2_HexNAc_2_Fuc_1_ N-glycans and exemplifies the positive effect of pyridylamination in determining the occurrence and even, based on relative intensities of the *m*/*z* 300 and 446 positive mode fragment ions, the type of core fucosylation

A first step in a detailed global glycomic analysis is to consider the means of releasing the oligosaccharide chains from the proteins or lipids. For cleavage from polypeptides, both chemical and enzymatic approaches are possible. Whereas the former (*e.g*., hydrazinolysis or β-elimination) can prove harsh and can affect labile residues on the analytes, the latter are restricted by enzyme specificities, which mean that some classes of glycans will not be released. For N-glycans, peptide:N-glycosidases (PNGases) are known from both bacterial and eukaryotic sources; most used are the commercially-available ones from *Flavobacterium* (PNGase F) and almond (PNGase A). The familiar PNGase F is unable to cleave core α1-3-fucosylated N-glycans [46] while being less affected by the peptide length; the opposite is true for PNGase A, which can remove such glycans, but optimally only from small peptides. Therefore, as core α1-3-fucose is common in invertebrates and in some protists, best results are obtained if both enzymes are employed sequentially. Indeed, we generally proteolyse samples and then treat the glycopeptides first with PNGase F to release the bulk of the N-glycans (*e.g*., oligomannosidic and core α1-6-fucosylated structures) prior to using PNGase A to cleave the remaining ones carrying the core α1-3-fucose. This selective release results in a further deconvolution of complex glycomes even before the enrichment into neutral and anionic pools (Fig. 2). Whereas new PNGases have been recently discovered with broader substrate specificities [47–49], a universal O-glycanase is yet to be found.

Although glycans are often analysed as mixtures, it is undoubtedly optimal if a suitable fractionation method is used to overcome suppression of glycans of low abundance. Normal phase, reversed phase and graphitised carbon materials are commonly used in glycan purification, either to initially ‘clean-up’ a glycan preparation, crudely separate glycan pools or purify into fractions with each containing a low number of glycan species. Graphitised carbon has proven to be an invaluable solid phase for the enrichment of anionic oligosaccharides, whether the anionic moiety be sialic acid, glucuronate, sulphate or phosphate [4, 12, 50, 51]; however, dominant monoanionic glycans are also found in the ‘neutral’ fraction. Typically, we elute the neutral-enriched fraction with 40 % acetonitrile prior to the ‘acidic-enriched’ pool with 0.1 % trifluoroacetic acid and these pools are often then ready for mass spectrometric analyses, although occasionally a subsequent reversed-phase step may be required as in a recent study on a marine snail [14]. The next option is whether to analyse free, reduced, permethylated or fluorescently-labelled N-glycans. In our own studies, we observe that labelling with 2-aminopyridine, which facilitates the use of reversed phase HPLC columns, also improves the ionisation of the oligosaccharides and the fragmentation of the core region (thus allowing direct demonstration of core fucosylation; Fig. 2).

During the analysis of fluorescently-labelled N-glycans, classical C18 columns (*e.g.,* Hypersil) are commonly employed [52], but in a study on the free-living nematode *Pristionchus pacificus* we compared Hypersil C18 with two fused-core reversed-phase resins (RP-amide and Kinetex XB-C18). Thereby, differences in the retention of phosphorylcholine-modified glycans were observed; for the standard C18 material, these glycans were rather highly-retained, whereas on the fused core columns they eluted earlier and, in general, isomers were more finely separated [19]. This means that, for a typical polishing step with C18 reversed-phase resin, sufficient organic solvent must be applied to elute phosphorylcholine-modified glycans; otherwise these glycans may be lost. In contrast, N-glycans modified with phosphoethanolamine, methylphosphate or sulphate elute early on a standard C18 column [16, 53]. While glycans with multiple such residues are poorly resolved/retained on reversed phase columns, the power of this method is to resolve isomers of neutral and mono-anionic glycans; this is especially useful when the target is to distinguish antennal or core modifications.

Normal phase resins are also frequently used for N-glycan analyses and their application for size fractionation is reminiscent of the gel filtration methods (*e.g*., Bio-Gel P4) common in the 1980’s and 1990’s for exoglycosidase-based glycan sequencing [54]. Interestingly, sulphate not only leads to earlier retention on reversed phase columns, but also on normal phase [13]. On the other hand, a ‘mixed’ hydrophilic interaction/anion exchange resin (HIAX; Dionex AS11) is an excellent means of separating by size as well as by increasing numbers of anionic residues, regardless of type [4, 12, 55]. For samples particularly complex in terms of numbers of glycans of different mass, 2D-HPLC (*e.g*., with normal phase as a first or second separation step) can also be used to reduce sample complexity prior to mass spectrometry before and after enzymatic or chemical treatments.

## Fast atom bombardment spectrometry

Historically, the use of FAB-MS was extremely useful in N-glycan analysis; in the 1990’s, this method was used to analyse oligosaccharides from nematodes carrying phosphorylcholine [36, 56] as well as glycosylphosphatidylinositol anchors from eukaryotes modified with phosphoethanolamine [57]. As the glycans were commonly derivatised by permethylation, subsequent extraction procedures can result in the loss of the polar ionically-modified glycans. Thus, the zwitterionic glycans were only detected either by permethylation after hydrofluoric acid (HF) treatment, which removes the phosphorylcholine moieties, or by peracetylation which is suitable for lower mass glycans: both approaches were employed on N-glycans from nematode parasites [36, 56]. Thus, in part, the positions of the phosphorylcholine modifications could only be inferred.

On the other hand, the presence of aminoethylphosphonate on locust N-glycans was fully compatible with standard FAB-MS [18], with the 6-substitution of mannose or GlcNAc being shown by displacement with a perdeuteromethyl group (^31^P and ^1^H-NMR were also employed in that study). Positive and negative ion mode FAB-MS also revealed the presence of aminoethylphosphonate or its *N-*methylated derivative on mollusc glycolipids [43, 58]. As for studies on phosphorylcholine-modified glycoconjugates, HF also removed the phosphonate to yield the underlying neutral structure. Seemingly sulphated glycans have been less studied using FAB-MS, but examples include repeating units of the dermatan sulphate of a marine ascidian [59] and of the chondroitin sulphate from squid [60] as well as detection of sulphated *N-*glycolylneuraminic acid in sea urchin eggs [61]. Furthermore, FAB-MS was one of a number of methods used to show the presence of methylphosphate on *Dictyostelium* N-glycans [62].

## Electrospray mass spectrometry

Currently ESI-MS and MALDI-TOF MS are the most widely-used mass spectrometric methods for glycan analysis. ESI-MS is often considered ‘softer’ than MALDI-TOF MS [45] and is frequently used ‘on-line’ with liquid chromatography, but tends to produce multiply-charged ions [63]. It appears that there is only one study on the analysis of phosphorylcholine-modified N-glycans by LC-ESI-MS; on-line porous-graphitised carbon separation of pyridylaminated oligosaccharides from *Ascaris suum* thereby revealed a set of eleven zwitterionic N-glycans which were all sensitive to hydrofluoric acid treatment; as a signature MS/MS fragment ion for nematode antennal phosphorylcholine modifications, *m*/*z* 369 was detected which corresponds to PC_1_HexNAc_1_ [64]*.* ESI-MS was also used to analyse an unusual phosphoethanolamine-modified N-glycan originating from *Campylobacter* [65]. In terms of O-glycans, ESI-MS has revealed the presence of phosphorylcholine and phosphoethanolamine linked to insect O-glycans [12, 66] as well as of aminoethylphosphonate on a jellyfish O-glycan [44].

Examples of invertebrate phosphorylated and sulphated protein-linked glycans analysed, at least in part, by ESI-MS include unusual phosphoglycans from *Trypanosoma cruzi* and *Leishmania* [67–69], a phosphodiester-linked disaccharide from *Dictyostelium* [70] and fragments of a sulphated polysaccharide from a starfish [71]. Whereas underivatised sulphated O-glycans from insects and an example sulphated N-glycan from an oyster were analysed by PGC-ESI-MS/MS, a modified post-permethylation clean-up procedure allowed detection of sulphated N-glycans in mosquito using nanospray mass spectrometry [12, 13]. NSI-MS following permethylation was also used to detect sialic acid on N-glycans from *Drosophila* and glucuronic acid on O-glycans from both *Drosophila* and mosquitoes [12, 21], while ESI-IT-MS was used to define methylated glucuronic acid substitutions of antennal fucose on N-glycans derived from the shell-forming fluid of a mussel [28]. In the case of another mollusc, aspects of the structure of the GlcA(MeHex)Fuc branches and the methylaminoethylphosphonate-modified N-glycans, as well as zwitterionic and anionic O-glycans, of a marine snail were revealed by PGC-ESI-MS/MS [14].

## Matrix-assisted laser-desorption/ionisation mass spectrometry

MALDI-TOF MS is arguably the most robust, versatile and flexible method for glycomic analysis, although the ionisation is often considered relatively harsh; it tends to produce singly-charged ions in the mass range relevant to glycan analysis, which eases interpretation. It can either be performed on free glycans or ones in permethylated form or those derivatised with a fluorescent label at the reducing terminus [72]. While modified post-derivatisation strategies allow MALDI-TOF MS of sulphated glycans in their permethylated form [73], fluorescent labelling of glycans with, *e.g*., 2-aminopyridine or 2-aminobenzaminidine, facilitates HPLC methods which can then be used ‘off-line’ with MALDI-TOF MS. As mentioned above, in the case of 2-aminopyridine, labelling also increases sensitivity in the mass spectrometer and yields intense *Y-*fragments of protonated quasimolecular ions, which aid identification of core modifications without affecting the ability to observe *B*-ions resulting from antennal losses. Over the years various matrices have been proposed for glycan analysis and currently we use 6-aza-2-thiothymine (ATT) in both positive and negative ion modes.

**Fig. 3:**
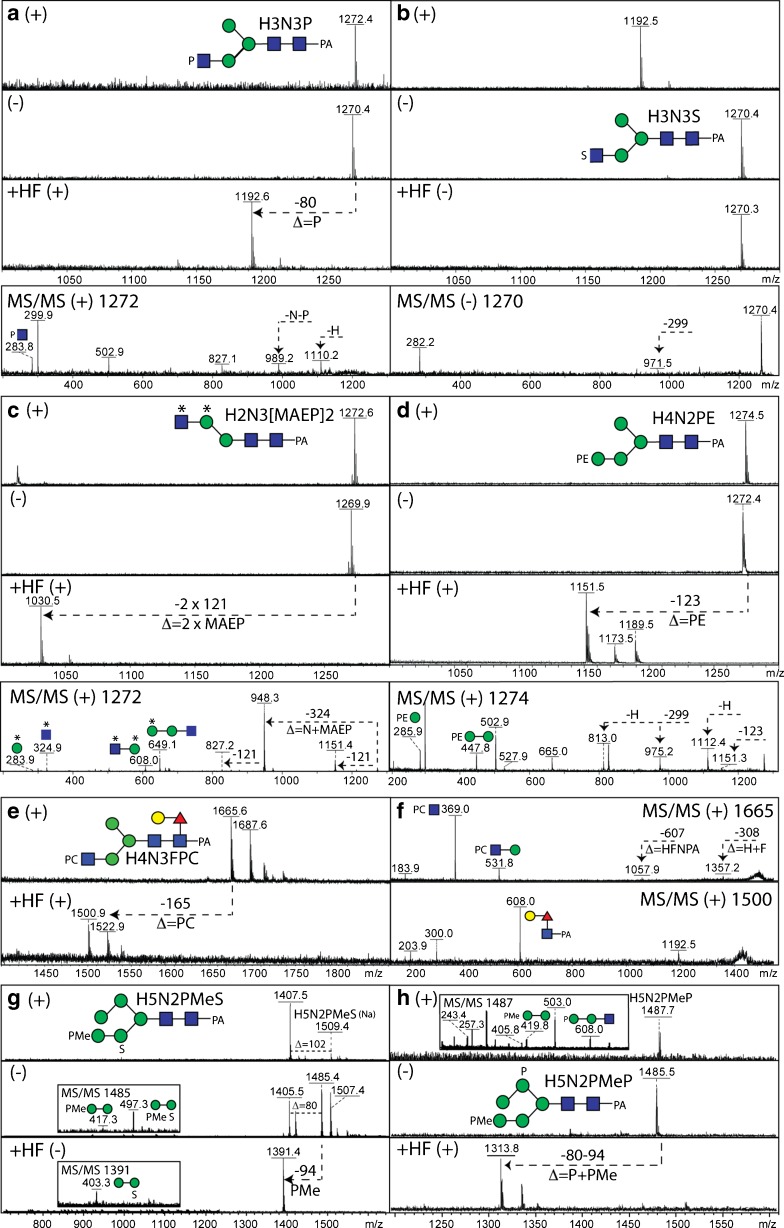
Comparative mass spectrometry of N-glycans modified by phosphoesters and sulphate. Example positive and negative ion mode MALDI-TOF MS and MS/MS (also after hydrofluoric acid treatment) of N-glycans from invertebrate and protist organisms. **(a-c)** Comparison of the positive (+) and negative (−) ion mode MALDI-TOF MS spectra before and after hydrofluoric acid (HF) treatment of isobaric N-glycans modified with either phosphate (P), sulphate (S) or methylaminoethylphosphonate (MAEP or *) from two marine organisms together with a relevant MS/MS spectrum of either the *m*/*z* 1270 or 1272 [M-H]^−^ or [M + H]^+^ quasimolecular ions. **(d)** Positive (+) and negative (−) ion mode MALDI-TOF MS spectra before and after hydrofluoric acid treatment of a *Trichomonas vaginalis* N-glycan (Tv2 strain) with the corresponding MS/MS spectrum of *m*/*z* 1274. **(e, f)** Positive ion mode MALDI-TOF MS and MS/MS of a nematode N-glycan modified with phosphorylcholine (PC) before and after hydrofluoric acid treatment. **(g, h)** Comparison of the positive (+) and negative (−) ion mode MALDI-TOF MS spectra before and after hydrofluoric acid (HF) treatment of isobaric N-glycans modified with methylphosphate (PMe) and phosphate or sulphate from *Dictyostelium* with relevant MS/MS spectra of either the *m*/*z* 1485 or 1487 quasimolecular ions. Further structural details were in each case proven by enzymatic digestion. The data in **a** are unpublished, whereas those in panels **b-h** underlie assignments in previous publications on *Volvarina rubella* (**b** and **c**; marine snail), *Trichomonas vaginalis* (**d**; protist), *Caenorhabditis elegans* (**e** and **f**; nematode) and *Dictyostelium discoideum* (**g** and **h**; slime mould) [4, 14, 16, 74]

Considering the individual types of modifications, the zwitterionic character of phosphorylcholine ensures that glycans which carry this moiety are rather easily detected in the positive ion mode, with the *m*/*z* 328 or 369 ions (PC_1_Hex_1_ and PC_1_HexNAc_1_) being strong characteristic antennal MS/MS fragments as observed, respectively, for fungal and nematode N-glycans [17, 19]. Indeed, even with pyridylaminated glycans for which the core region is generally well observed in MS/MS, antennal PC_1_Hex(NAc)_1_ fragments dominate the spectrum and so information regarding the core is only inferred by fragment ions corresponding to the loss of the core (*e.g*., loss of 299, 445 or 607 for GlcNAc_1_Fuc_0–1_Gal_0–1_-PA; see Fig. 3e and f and Refs. [19, 74]). In a marine snail, we have also found phosphorylcholine substituting galactose linked to core α1-6-fucose residues [14].

Although hydrofluoric acid treatment, which removes phosphorylcholine (amongst other phosphoesters as well as galactofuranose and some fucose linkages), tends to reduce the signal of low abundance glycans, in part due to the loss of the well-ionisable zwitterionic modification, it does then allow the core *Y*-fragments (*e.g*., *m*/*z* 300, 446 or 608; GlcNAc_1_Fuc_0–1_Gal_0–1_-PA) to be observed if the glycan is derivatised at the reducing terminus. In the case of the purified *Echinococcus granulosus* Ag5 antigen, MALDI-TOF MS of the glycopeptides also revealed the presence of phosphorylcholine-modified oligosaccharides with the choice of 6-aza-2-thiothymine as matrix proving important [15]. It is to be noted that if a whole glycome or an individual HPLC fraction contains a phosphorylcholine-modified N-glycan, ‘contamination’ of the MS/MS spectra of other glycans can occur, which emphasises the need for adequate purification.

In terms of phosphoethanolamine modifications, off-line LC-MALDI-MS studies on *Trichomonas vaginalis* revealed early-eluting RP-HPLC fractions in some strains which contained glycans with a modification of 123 Da [16]. These glycans were observed not only in positive ion mode, as for phosphorylcholine, but also ionised well in the negative mode. The key positive mode fragments of *m*/*z* 286 and 448 are consistent with a Hex_1-2_PE_1_ motif [16], which is lost upon treatment with HF (Fig. 3d). We have recently also found, using off-line LC-MALDI-TOF MS, phosphoethanolamine linked to mannose as a component of *Penicillium* N-glycans [17], while methylaminoethylphosphonate, which is also HF sensitive (Fig. 3c), is found on N-glycans of a marine snail, *Volvarina rubella* [14]*.*

Also, in our recent studies, we have revealed sulphation on a range of invertebrate N-glycans, relying on a number of approaches to show that the 80 Da modification of these oligosaccharides is indeed sulphate and not phosphate; generally, mass accuracies of many instruments (other than FT-MS) are insufficient to distinguish phosphate and sulphate (Δ = 0.01 Da), but there are a number of differences regarding their properties. First, phosphorylated glycans ‘fly’ well in both positive and negative ion modes, whereas the detection of sulphated glycans is often impeded by ‘in source’ loss of sulphate, an effect most obvious in the positive ion mode. This can, though, depend on the choice of matrix. Using 2,5-dihydrobenzoic acid (DHB), sodiated adducts of intact sulphated glycans are detected; with ATT, traces of these [M + Na]^+^ ions can be observed in positive mode, whereas very good MS and MS/MS spectra can be obtained for the intact glycan in negative mode [13]. Second, phosphate (also methylphosphate, but not sulphate) is sensitive to hydrofluoric acid treatment resulting in a shift of 80 or 94 Da (Fig. 3a, b, g and h), whereas methanolic HCl can be used to remove sulphate at least to some extent under conditions which do not result in loss of other residues. We have applied these methods to example slime mould, insect and mollusc N-glycans [4, 12, 14]. Although there are commercially-available sulphatases, we have yet to find a source which is compatible with a MALDI-TOF-based digestion assay; in contrast, standard phosphatases are ‘MALDI-compatible’. Both phosphate and sulphate (like methylation) tend to block any enzymatic digestion of the residue to which they are attached.

For the analysis of glycomes of various species, we used HIAX, which fractionates not only due to size, but also on the basis of the number of charged residues, with neutral glycans eluting earliest and those with multiple anionic groups last. Thus, we could separate a range of sulphated N-glycans, some also containing glucuronic acid, from *Anopheles* as well as N-glycans from *Dictyostelium* carrying phosphate/methylphosphate 6-linked to mannose or sulphated oyster N-glycans [4, 12, 13, 53]*.* This overcame the problem that such glycans were early-eluting and not well-resolved when fractionated by RP-HPLC, particularly when they contained more than one anionic moiety. Interestingly, a phosphorylated Hex_5_HexNAc_2_PMeP glycan (for MS data, see Fig. 3h) eluted slightly earlier than the corresponding isobaric sulphated form on HIAX [4]. Furthermore, in the case of oyster plasma N-glycans, sulphation was observed to reduce retention time on both RP- and NP-HPLC; however, the sulphated glycans accounting for about half the structures, some also carrying the sulphate in the context of a blood group A modification, could be analysed by MS and MS/MS in negative ion mode with the monosulphated being detected as [M-H]^−^ and disulphated as [M-2H + Na]^−^ ions [13]. A mix of NP-HPLC, MALDI-TOF MS and ESI-MS has been recently applied by others to demonstrate sulphation of N-glycans from a lepidopteran species [75].

In the case of glucuronic acid, its mass is the same as a methylated hexose (*Δm*/*z* 176) and so it is important to distinguish these units from each other. Fortunately, not only are glucuronylated N-glycans enriched in the acidic fraction eluted from graphitised carbon, they ionise well in both positive and negative ion modes of MALDI-TOF MS; fragmentation in positive ion mode is effective as shown for mosquito N-glycans [12] as well as for glucuronylated antennae from a marine snail [14]. While sialic acid modifications can be detected by MALDI-TOF MS, with the potential to stabilise these residues in a linkage-specific manner [76], we have not yet detected sialic acids in our own studies on N-glycans derived from insects, nematodes, molluscs or protists other than in obvious food or media components.

A key part of our strategy is that exoglycosidases or chemical treatments are used to gain extra information, in addition to the retention time and MS/MS data, regarding the structure of an N-glycan. Thereby, we have also partly extended the tools available by having our own in-house recombinant exoglycosidases, such as galactosidases and hexosaminidases [77, 78], in addition to using those from commercial sources. However, the background contaminations in some enzyme preparations (*e.g.,* β-glucuronidase or α-galactosidase) can make subsequent MS measurements difficult and so low-abundance structures can be refractory to further analyses (only with reasonable amounts can reinjection onto HPLC be performed). A further challenge is that steric hindrance, a lack of standards or the inability to remove certain modifications (*e.g.,* methylation) can complicate the interpretation. Nevertheless, even pyridylaminated glycans of low abundance (*e.g.,* 2 pmol in an HPLC fraction) can be structurally defined using our approach by MS/MS before and after enzymatic and chemical incubation without further purification.

## Conclusion

Glycan analysis remains a challenge, especially for unknown glycans in low amount from novel sources; current glycoinformatic tools are very much mammalian-centered and ‘in silico’ annotations are not appropriate for the analysis of N-glycans from invertebrate or protist sources. Furthermore, analytical methods requiring a larger amount of material (NMR or GC-MS) cannot be performed on low abundance isomers and so one must rely on robust, sensitive procedures in which complex mixtures of glycans are avoided. This may, depending on the purity of the glycan sample, require one or two steps of solid-phase extraction followed by on-line or off-line fractionation in combination with mass spectrometry. Ideally, orthogonal methods (more than one type of mass spectrometry, chemical or enzymatic treatments) are used. These concepts have formed our own analytical workflows and the results of our recent studies highlight the extreme variability of N-glycan structures in invertebrates and protists; thereby, we observe that anionic or zwitterionic modifications are often significantly present. Thus, tailoring the methods to take this into account, one should always expect the unexpected when exploring the glycouniverse.

## References

[1] Kollmann K, Pohl S, Marschner K, Encarnacao M, Sakwa I, Tiede S, Poorthuis BJ, Lubke T, Muller-Loennies S, Storch S, Braulke T (2010). Mannose phosphorylation in health and disease. Eur. J. Cell Biol..

[2] Fiete D, Srivastava V, Hindsgaul O, Baenziger JU (1991). A hepatic reticuloendothelial cell receptor specific for SO_4_-4GalNAcβ1,4GlcNAcβ1,2Manα that mediates rapid clearance of lutropin. Cell.

[3] Kizuka Y, Oka S (2012). Regulated expression and neural functions of human natural killer-1 (HNK-1) carbohydrate. Cell. Mol. Life Sci..

[4] Hykollari A, Balog CI, Rendić D, Braulke T, Wilson IBH, Paschinger K (2013). Mass spectrometric analysis of neutral and anionic N-glycans from a *Dictyostelium discoideum* model for human congenital disorder of glycosylation CDG IL. J. Proteome Res..

[5] Takashiba M, Chiba Y, Jigami Y (2006). Identification of phosphorylation sites in N-linked glycans by matrix-assisted laser desorption/ionization time-of-flight mass spectrometry. Anal. Chem..

[6] Pomin VH, Mourão PA (2008). Structure, biology, evolution, and medical importance of sulfated fucans and galactans. Glycobiology.

[7] Bülow HE, Hobert O (2006). The molecular diversity of glycosaminoglycans shapes animal development. Annu. Rev. Cell Dev. Biol..

[8] Miyata S, Sato C, Kitamura S, Toriyama M, Kitajima K (2004). A major flagellum sialoglycoprotein in sea urchin sperm contains a novel polysialic acid, an α2,9-linked poly-N-acetylneuraminic acid chain, capped by an 8-O-sulfated sialic acid residue. Glycobiology.

[9] Shimojima M (2011). Biosynthesis and functions of the plant sulfolipid. Prog. Lipid Res..

[10] Freeze HH (1985). Mannose 6-sulfate is present in the N-linked oligosaccharides of lysosomal enzymes of *Dictyostelium*. Arch. Biochem. Biophys..

[11] Varki A, Cummings RD, Aebi M, Packer NH, Seeberger PH, Esko JD, Stanley P, Hart G, Darvill A, Kinoshita T, Prestegard JJ, Schnaar RL, Freeze HH, Marth JD, Bertozzi CR, Etzler ME, Frank M, Vliegenthart JF, Lutteke T, Perez S, Bolton E, Rudd P, Paulson J, Kanehisa M, Toukach P, Aoki-Kinoshita KF, Dell A, Narimatsu H, York W, Taniguchi N, Kornfeld S (2015). Symbol Nomenclature for Graphical Representations of Glycans. Glycobiology.

[12] Kurz S, Aoki K, Jin C, Karlsson NG, Tiemeyer M, Wilson IB, Paschinger K (2015). Targetted release and fractionation reveal glucuronylated and sulphated N- and O-glycans in larvae of dipteran insects. J. Proteomics.

[13] Kurz S, Jin C, Hykollari A, Gregorich D, Giomarelli B, Vasta GR, Wilson IBH, Paschinger K (2013). Haemocytes and plasma of the eastern oyster (*Crassostrea virginica*) display a diverse repertoire of sulphated and blood group a-modified N-glycans. J. Biol. Chem..

[14] Eckmair B., Jin C., Abed-Navandi D., Paschinger K.: Multi-step fractionation and mass spectrometry reveals zwitterionic and anionic modifications of the N- and O-glycans of a marine snail. Mol Cell Proteomics. **15**, 573–597 (2016). doi:10.1074/mcp.M115.051573 10.1074/mcp.M115.051573 PMC4739674 26598642

[15] Paschinger K, Gonzalez-Sapienza GG, Wilson IBH (2012). Mass spectrometric analysis of the immunodominant glycan epitope of *Echinococcus granulosus* antigen Ag5. Int. J. Parasitol..

[16] Paschinger K, Hykollari A, Razzazi-Fazeli E, Greenwell P, Leitsch D, Walochnik J, Wilson IBH (2012). The N-glycans of *Trichomonas vaginalis* contain variable core and antennal modifications. Glycobiology.

[17] Hykollari A., Eckmair B., Voglmeir J., Jin C., Yan S., Vanbeselaere J., Razzazi-Fazeli E., Wilson I.B.H., Paschinger K.: More than just oligomannose: an N-glycomic comparison of *Penicillium* species. Mol Cell Proteomics **15**, 73-92 (2016). doi:10.1074/mcp.M1115.055061 10.1074/mcp.M115.055061 PMC4700520 26515459

[18] Hård K, Van Doorn JM, Thomas-Oates JE, Kamerling JP, Van der Horst DJ (1993). Structure of the Asn-linked oligosaccharides of apolipophorin III from the insect *Locusta migratoria*. Carbohydrate- linked 2-aminoethylphosphonate as a constituent of a glycoprotein. Biochemistry.

[19] Yan S, Wilson IBH, Paschinger K (2015). Comparison of RP-HPLC modes to analyse the N-glycome of the free-living nematode *Pristionchus pacificus*. Electrophoresis.

[20] Eugenia Giorgi, M., de Lederkremer, R.M.: Trans-sialidase and mucins of *Trypanosoma cruzi*: an important interplay for the parasite. Carbohydr. Res. **346**, 1389–1393 (2011). 10.1016/j.carres.2011.04.006 21645882

[21] Aoki K, Perlman M, Lim JM, Cantu R, Wells L, Tiemeyer M (2007). Dynamic developmental elaboration of N-linked glycan complexity in the *Drosophila melanogaster* embryo. J. Biol. Chem..

[22] Koles K, Irvine KD, Panin VM (2004). Functional characterization of *drosophila* sialyltransferase. J. Biol. Chem..

[23] Repnikova E, Koles K, Nakamura M, Pitts J, Li H, Ambavane A, Zoran MJ, Panin VM (2010). Sialyltransferase regulates nervous system function in *Drosophila*. J Neurosci.

[24] Miyata S, Sato C, Kumita H, Toriyama M, Vacquier VD, Kitajima K (2006). Flagellasialin: a novel sulfated α2,9-linked polysialic acid glycoprotein of sea urchin sperm flagella. Glycobiology.

[25] Toyoda H, Kinoshita-Toyoda A, Seleck SB (2000). Structural analysis of glycosaminoglycans in *Drosophila* and *Caenorhabditis elegans* and demonstration that tout-velu, a drosophila gene related to EXT tumour suppressors, affects heparan sulphate *in vivo*. J. Biol. Chem..

[26] Weske B, Dennis RD, Helling F, Keller M, Nores GA, Peter-Katalinic J, Egge H, Dabrowski U, Wiegandt H (1990). Glycosphingolipids in insects. Chemical structures of two variants of a glucuronic-acid-containing ceramide hexasaccharide from a pupae of *Calliphora vicina* (insecta: diptera), distinguished by a *N*-acetylglucosamine-bound phosphoethanolamine sidechain. Eur. J. Biochem..

[27] Breloy I, Schwientek T, Lehr S, Hanisch FG (2008). Glucuronic acid can extend O-linked core 1 glycans, but it contributes only weakly to the negative surface charge of *Drosophila melanogaster* Schneider-2 cells. FEBS Lett..

[28] Zhou H, Hanneman AJ, Chasteen ND, Reinhold VN (2013). Anomalous N-glycan structures with an internal fucose branched to GlcA and GlcN residues isolated from a mollusk shell-forming fluid. J. Proteome Res..

[29] Bergwerff AA, Van Dam GJ, Rotmans JP, Deelder AM, Kamerling JP, Vliegenthart JFG (1994). The immunologically reactive part of immunopurified circulating anodic antigen from *Schistosoma mansoni* is a threonine- linked polysaccharide consisting of →6)-(β-D-Glc*p*A-(1 → 3))-β-D-Gal*p*NAc-(1 → repeating units. J. Biol. Chem..

[30] Parsons LM, Mizanur RM, Jankowska E, Hodgkin J, O'Rourke D, Stroud D, Ghosh S, Cipollo JF (2014). *Caenorhabditis elegans* bacterial pathogen resistant *bus-4* mutants produce altered mucins. PLoS One.

[31] Serino L, Virji M (2002). Genetic and functional analysis of the phosphorylcholine moiety of commensal *Neisseria* lipopolysaccharide. Mol. Microbiol..

[32] Mackinnon FG, Cox AD, Plested JS, Tang CM, Makepeace K, Coull PA, Wright JC, Chalmers R, Hood DW, Richards JC, Moxon ER (2002). Identification of a gene (*lpt-3*) required for the addition of phosphoethanolamine to the lipopolysaccharide inner core of *Neisseria meningitidis* and its role in mediating susceptibility to bactericidal killing and opsonophagocytosis. Mol. Microbiol..

[33] Pineda MA, Lumb F, Harnett MM, Harnett W (2014). ES-62, a therapeutic anti-inflammatory agent evolved by the filarial nematode *Acanthocheilonema viteae*. Mol. Biochem. Parasitol..

[34] Kinoshita T (2014). Biosynthesis and deficiencies of glycosylphosphatidylinositol. Proc Jpn Acad Ser B Phys Biol Sci.

[35] Unkefer CJ, Jackson C, Gander JE (1982). The 5-O-β-D-galactofuranosyl-containing glycopeptide from *Penicillium charlesii.* Identification of phosphocholine attached to C-6 of mannopyranosyl residues of the mannan region. J. Biol. Chem..

[36] Haslam SM, Houston KM, Harnett W, Reason AJ, Morris HR, Dell A (1999). Structural studies of N-glycans of filarial parasites. Conservation of phosphorylcholine-substituted glycans among species and discovery of novel chito-oligomers. J Biol Chem.

[37] Wilson, I.B.H., Paschinger, K.: Sweet secrets of a therapeutic worm: Mass spectrometric N-glycomic analysis of *Trichuris suis*. Anal. Bioanal. Chem.**408**, 461–471 (2016). 10.1007/s00216-015-9154-8 PMC4712359 26650734

[38] Simenel C, Coddeville B, Delepierre M, Latgé JP, Fontaine T (2008). Glycosylinositolphosphoceramides in *Aspergillus fumigatus*. Glycobiology.

[39] Sugita M, Fujii H, Inagaki F, Suzuki M, Hayata C, Hori T (1992). Polar glycosphingolipids in annelida. A novel series of glycosphingolipids containing choline phosphate from the earthworm, *Pheretima hilgendorf*. J. Biol. Chem..

[40] Friedl CH, Lochnit G, Zähringer U, Bahr U, Geyer R (2003). Structural elucidation of zwitterionic carbohydrates derived from glycosphingolipids of the porcine parasitc nematode *Ascaris suum*. Biochem. J..

[41] Maes E, Garenaux E, Strecker G, Leroy Y, Wieruszeski JM, Brassart C, Guerardel Y (2005). Major O-glycans from the nest of *Vespula germanica* contain phospho-ethanolamine. Carbohydr. Res..

[42] Seppo A, Moreland M, Schweingruber H, Tiemeyer M (2000). Zwitterionic and acidic glycosphingolipids of the *Drosophila melanogaster* embryo. Eur. J. Biochem..

[43] Hayashi A, Matsubara T (1989). A new homolog of phosphonoglycosphingolipid, N-methylaminoethylphosphonyltrigalactosylceramide. Biochim. Biophys. Acta.

[44] Urai M, Nakamura T, Uzawa J, Baba T, Taniguchi K, Seki H, Ushida K (2009). Structural analysis of O-glycans of mucin from jellyfish (*Aurelia aurita*) containing 2-aminoethylphosphonate. Carbohydr. Res..

[45] Zaia J (2010). Mass spectrometry and glycomics. OMICS.

[46] Tretter V, Altmann F, März L (1991). Peptide-*N*^4^-(*N*-acetyl-β-glucosaminyl)asparagine amidase F cannot release glycans with fucose attached α1 → 3 to the asparagine-linked *N*-acetylglucosamine residue. Eur. J. Biochem..

[47] Lee KJ, Gil JY, Kim SY, Kwon O, Ko K, Kim DI, Kim DK, Kim HH, Oh DB (2014). Molecular characterization of acidic peptide:N-glycanase from the dimorphic yeast *Yarrowia lipolytica*. J. Biochem..

[48] Wang T, Cai ZP, Gu XQ, Ma HY, Du YM, Huang K, Voglmeir J, Liu L (2014). Discovery and characterization of a novel extremely acidic bacterial N-glycanase with combined advantages of PNGase F and A. Biosci. Rep..

[49] Sun G, Yu X, Bao C, Wang L, Li M, Gan J, Qu D, Ma J, Chen L (2015). Identification and characterization of a novel prokaryotic peptide: N-glycosidase from *Elizabethkingia meningoseptica*. J. Biol. Chem..

[50] Packer NH, Lawson MA, Jardine DR, Redmond JW (1998). A general approach to desalting oligosaccharides released from glycoproteins. Glycoconj. J..

[51] Chu CS, Ninonuevo MR, Clowers BH, Perkins PD, An HJ, Yin H, Killeen K, Miyamoto S, Grimm R, Lebrilla CB (2009). Profile of native N-linked glycan structures from human serum using high performance liquid chromatography on a microfluidic chip and time-of-flight mass spectrometry. Proteomics.

[52] Tomiya N, Kurono M, Ishihara H, Tejima S, Endo S, Arata Y, Takahashi N (1987). Structural analysis of N-linked oligosaccharides by a combination of glycopeptidase, exoglycosidases, and high-performance liquid chromatography. Anal. Biochem..

[53] Hykollari A, Dragosits M, Rendić D, Wilson IBH, Paschinger K (2014). N-glycomic profiling of a glucosidase II mutant of *Dictyostelium discoideum* by “off-line” liquid chromatography and mass spectrometry. Electrophoresis.

[54] Kobata A (1979). Use of endo- and exoglycosidases for structural studies of glycoconjugates. Anal. Biochem..

[55] Neville DC, Dwek RA, Butters TD (2009). Development of a single column method for the separation of lipid- and protein-derived oligosaccharides. J. Proteome Res..

[56] Morelle W, Haslam SM, Olivier V, Appleton JA, Morris HR, Dell A (2000). Phosphorylcholine-containing N-glycans of *Trichinella spiralis*: identification of multiantennary lacdiNAc structures. Glycobiology.

[57] Roberts WL, Santikarn S, Reinhold VN, Rosenberry TL (1988). Structural characterization of the glycoinositol phospholipid membrane anchor of human erythrocyte acetylcholinesterase by fast atom bombardment mass spectrometry. J. Biol. Chem..

[58] Araki S, Yamada S, Abe S, Waki H, Kon K, Itonori S, Sugita M, Ando S: (2001). Characterization of a novel triphosphonooctaosylceramide from the eggs of the sea hare, *Aplysia kurodai*. J. Biochem..

[59] Mourão PA, Pavão MS, Mulloy B, Wait R (1997). Chondroitin ABC lyase digestion of an ascidian dermatan sulfate. Occurrence of unusual 6-O-sulfo-2-acetamido-2-deoxy-3-O-(2-O-sulfo-α-L-idopyranosyluronic acid)-β-D-galactose units. Carbohydr. Res..

[60] Kinoshita-Toyoda A, Yamada S, Haslam SM, Khoo KH, Sugiura M, Morris HR, Dell A, Sugahara K (2004). Structural determination of five novel tetrasaccharides containing 3-O-sulfated D-glucuronic acid and two rare oligosaccharides containing a β-D-glucose branch isolated from squid cartilage chondroitin sulfate E. Biochemistry.

[61] Kitazume S, Kitajima K, Inoue S, Haslam SM, Morris HR, Dell A, Lennarz WJ, Inoue Y (1996). The occurrence of novel 9-O-sulfated N-glycolylneuraminic acid-capped α2 → 5-Oglycolyl-linked oligo/polyNeu5Gc chains in sea urchin egg cell surface glycoprotein. Identification of a new chain termination signal for polysialyltransferase. J. Biol. Chem.

[62] Couso R, van Halbeek H, Reinhold V, Kornfeld S (1987). The high mannose oligosaccharides of *Dictyostelium discoideum* glycoproteins contain a novel intersecting N-acetylglucosamine residue. J. Biol. Chem..

[63] Pabst M, Altmann F (2011). Glycan analysis by modern instrumental methods. Proteomics.

[64] Pöltl G, Kerner D, Paschinger K, Wilson IBH (2007). N-glycans of the porcine nematode parasite *Ascaris suum* are modified with phosphorylcholine and core fucose residues. FEBS J.

[65] Scott NE, Nothaft H, Edwards AV, Labbate M, Djordjevic SP, Larsen MR, Szymanski CM, Cordwell SJ (2012). Modification of the *Campylobacter jejuni* N-linked glycan by EptC protein-mediated addition of phosphoethanolamine. J. Biol. Chem..

[66] Gaunitz S, Jin C, Nilsson A, Liu J, Karlsson NG, Holgersson J (2013). Mucin-type proteins produced in the *Trichoplusia ni* and *Spodoptera frugiperda* insect cell lines carry novel O-glycans with phosphocholine and sulfate substitutions. Glycobiology.

[67] Allen S, Richardson JM, Mehlert A, Ferguson MAJ (2013). Structure of a complex phosphoglycan epitope from gp72 of *Trypanosoma cruzi*. J. Biol. Chem..

[68] Macrae JI, Acosta-Serrano A, Morrice NA, Mehlert A, Ferguson MA (2005). Structural characterization of NETNES, a novel glycoconjugate in *Trypanosoma cruzi* epimastigotes. J. Biol. Chem..

[69] Ilg T, Craik D, Currie G, Multhaup G, Bacic A (1998). Stage-specific proteophosphoglycan from *Leishmania mexicana* amastigotes - structural characterization of novel mono-, di-, and triphosphorylated phosphodiester-linked oligosaccharides. J. Biol. Chem..

[70] Mreyen M, Champion A, Srinivasan S, Karuso P, Williams KL, Packer NH (2000). Multiple O-glycoforms on the spore coat protein SP96 in *Dictyostelium discoideum*. Fuc(α1–3)GlcNAc-α-1-P-Ser is the major modification. J. Biol. Chem..

[71] Gunaratne HM, Yamagaki T, Matsumoto M, Hoshi M (2003). Biochemical characterization of inner sugar chains of acrosome reaction-inducing substance in jelly coat of starfish eggs. Glycobiology.

[72] Harvey DJ (1999). Matrix-assisted laser desorption/ionization mass spectrometry of carbohydrates. Mass Spectrom. Rev..

[73] Yu SY, Wu SW, Hsiao HH, Khoo KH (2009). Enabling techniques and strategic workflow for sulfoglycomics based on mass spectrometry mapping and sequencing of permethylated sulfated glycans. Glycobiology.

[74] Yan S., Jin C., Wilson I.B.H., Paschinger K.: Comparisons of *Caenorhabditis* fucosyltransferase mutants reveal a multiplicity of isomeric N-glycan structures. J. Proteome Res. **14**, 5291–305 (2015). doi:10.1021/acs.jproteome.1025b00746 10.1021/acs.jproteome.5b00746 PMC4673604 26538210

[75] Cabrera G., Salazar V., Montesino R., Tambara Y., Struwe W.B., Lugo E.L., Harvey D.J., Antoine L., Rincon M., Domon B., Mendez Martinez M.D., Portela M., Gonzalez-Hernandez A., Triguero A., Duran R., Lundberg U., Vonasek E., Gonzalez L.J.: Structural characterization and biological implications of sulfated N-glycans in a serine protease from the neotropical moth *Hylesia metabus* (Cramer [1775]) (lepidoptera: saturniidae). Glycobiology. **26**, 230–250 (2016). doi:10.1093/glycob/cwv096 10.1093/glycob/cwv096 26537504

[76] Reiding KR, Blank D, Kuijper DM, Deelder AM, Wuhrer M (2014). High-throughput profiling of protein N-glycosylation by MALDI-TOF-MS employing linkage-specific sialic acid esterification. Anal. Chem..

[77] Dragosits M, Pflugl S, Kurz S, Razzazi-Fazeli E, Wilson IBH, Rendić D (2014). Recombinant Aspergillus β-galactosidases as a robust glycomic and biotechnological tool. Appl. Microbiol. Biotechnol..

[78] Dragosits M, Yan S, Razzazi-Fazeli E, Wilson IBH, Rendić D (2015). Enzymatic properties and subtle differences in the substrate specificity of phylogenetically distinct invertebrate N-glycan processing hexosaminidases. Glycobiology.

